# Effect of *ALDH1A1* Gene Knockout on Drug Resistance in Paclitaxel and Topotecan Resistant Human Ovarian Cancer Cell Lines in 2D and 3D Model

**DOI:** 10.3390/ijms23063036

**Published:** 2022-03-11

**Authors:** Marta Nowacka, Barbara Ginter-Matuszewska, Monika Świerczewska, Karolina Sterzyńska, Michał Nowicki, Radosław Januchowski

**Affiliations:** 1 Department of Histology and Embryology, Poznan University of Medical Sciences, Święcickiego 6 St., 61-781 Poznan, Poland; mswierczewska@ump.edu.pl (M.Ś.); k.olejniczak@ump.edu.pl (K.S.); mnowicki@ump.edu.pl (M.N.); 2 Department of Infectious Diseases, Hepatology and Acquired Immunodeficiency, Poznan University of Medical Sciences, 61-003 Poznan, Poland; bgintermatuszewska@ump.edu.pl; 3 Department of Anatomy and Histology, Collegium Medicum, University of Zielona Gora, Zyty 28 St., 65-046 Zielona Gora, Poland; r.januchowski@cm.uz.zgora.pl

**Keywords:** ovarian cancer, spheroids, cancer stem cells

## Abstract

Ovarian cancer is the most common cause of gynecological cancer death. Cancer Stem Cells (CSCs) characterized by drug transporters and extracellular matrix (ECM) molecules expression are responsible for drug resistance development. The goal of our study was to examine the role of aldehyde dehydrogenase 1A1 (ALDH1A1) expression in paclitaxel (PAC) and topotecan (TOP) resistant ovarian cancer cell lines. In both cell lines, we knocked out the *ALDH1A1* gene using the CRISPR/Cas9 technique. Additionally, we derived an ALDH1A1 positive TOP-resistant cell line with ALDH1A1 expression in all cells via clonal selection. The effect of *ALDH1A1* gene knockout or clonal selection on the expression of ALDH1A1, drug transporters (P-gp and BCRP), and ECM (COL3A1) was determined by Q-PCR, Western blot and immunofluorescence. Using MTT assay, we compared drug resistance in two-dimensional (2D) and three-dimensional (3D) cell culture conditions. We did not observe any effect of *ALDH1A1* gene knockout on *MDR1*/P-gp expression and drug resistance in the PAC-resistant cell line. The knockout of *ALDH1A1* in the TOP-resistant cell line resulted in a moderate decrease of BCRP and COL3A1 expression and weakened TOP resistance. The clonal selection of ALDH1A1 cells resulted in very strong downregulation of BCPR and COL3A1 expression and overexpression of *MDR1*/P-gp. This finally resulted in decreased resistance to TOP but increased resistance to PAC. All spheroids were more resistant than cells growing as monolayers, but the resistance mechanism differs. The spheroids’ resistance may result from the presence of cell zones with different proliferation paces, the density of the spheroid, ECM expression, and drug capacity to diffuse into the spheroid.

## 1. Introduction

Epithelial ovarian cancer (EOC) has the highest mortality rate of all gynecological malignancies [1]. Most patients are diagnosed with advanced disease with the presence of metastasis. The standard treatment includes debulking surgery followed by chemotherapy based on platinum (Cisplatin—CIS or carboplatin) and taxol (Paclitaxel—PAC) agents [2]. Despite a high response rate to first-line chemotherapy, most patients develop acquired drug resistance and disease relapse. In the case of platinum-sensitive patients, the same chemotherapy regimen is used in the second-line of chemotherapy. In the case of platinum-resistant patients, second-line chemotherapy includes other drugs like topotecan (TOP) and doxorubicin (DOX) [3]. Resistance to chemotherapy is a primary cause of treatment failure in ovarian cancer. There are many mechanisms involved in this phenomenon, including: genetic alterations, increased elimination of drugs, localization changes of therapeutics in the cells, dysregulation of cellular metabolism and altered drug metabolism, rapid repair of DNA and cellular membranes damage, as well as increased ability to tolerate these damages, oxidative stress minimalizing inhibition of apoptosis and epigenetic modifications [4]. However, the most crucial resistance mechanism at the cellular level is the active removal of cytotoxic agents from cancer cells through the overexpression of ATP-binding cassette (ABC) transporters [5,6,7]. The role of ABCB1 (P-glycoprotein—P-gp), ABCC2 (Multidrug Resistance-associated Protein 2—MRP2), and ABCG2 (Breast Cancer Resistant Protein—BCRP) is well-established in the development of drug resistance [5]. P-gp transports various drugs such as taxanes, anthracyclines, Vinca alkaloids, neutral and cationic organic compounds, digoxin and tyrosine kinase inhibitors [7]. The expression of P-gp is positively correlated with multidrug resistance in multiple cell lines and cancers [8]. MRP2 primarily transports drugs conjugated with sulfate, glucuronate, or glutathione, including methotrexate, arsenite, and cisplatin (CIS) [7]. BCRP is related to resistance to various agents, such as anthracyclines, mitoxantrone, and TOP [9]. Although much attention has been placed on resistance mechanisms related to cancer cells, increasing evidence indicates the role of the tumor microenvironment in resistance to chemotherapy [10]. The tumor microenvironment comprises cancer cells, tumor-associated fibroblast (CAFs), and immune cells [11] and can influence cancer cell survival via diverse mechanisms. Both CAFs and cancer cells produce many extracellular matrix components (ECM) (e.g., collagens). The dense cellular structure in the tumor and the high expression level of ECM molecules effectively result in limited drug delivery to cancer cells [12]. Some drugs like PAC can bind to ECM components, resulting in lower drug availability for cancer cells [13]. Eventually, the interaction between cancer cells and ECM increases intracellular signaling leading to cell adhesion-mediated drug resistance (CAM-DR) [14]. The latest mechanism seems to play a role in drug resistance not only in tumor tissue but also in cell culture in vitro. It has been observed that cancer cells cultured on collagen I-coated dishes were more resistant to chemotherapy [15]. Recently, we and others observed increased expression of ECM components in drug resistant ovarian cancer cell lines [16,17,18,19,20,21]. Especially we noted a high expression level of collagens in PAC- and TOP-resistant ovarian cancer cell lines [19,22]. As the mechanism of cancer drug resistance is very complex, different models have attempted to describe the development of drug resistance in cancer. Some studies have linked the development of drug resistance to the presence of a small fraction of tumor cells that are highly resistant to drugs and capable of surviving treatment. According to the cancer stem cells (CSC) hypothesis, these resistant cells are mostly cancer stem cells. CSCs are characterized by the expression of ABC drug transporters such as P-gp and BCRP, and other resistance mechanisms [23]. Among different markers of CSCs, the most universal among solid tumors is the expression of aldehyde dehydrogenase isoform 1A1—ALDH1A1 [24]. ALDH1A1 is also utilized as a marker of ovarian CSCs [25]. After drug treatment, only the CSCs (which express drug transporters) survive. These stem cells then divide and repopulate the tumor mass with stem cells and with differentiated but still drug-resistant cells that originate from the stem cells [26]. The ALDH1A1-positive cell population was observed in drug-resistant ovarian cancers and cell lines [25,27,28,29]. A large number of CSCs is also frequently described in the patient ascites [30]. An increased number of ALDH1 positive cells is negatively correlated with a patient’s survival [31,32]. Furthermore, the broad analysis of multiple ovarian cancer cell lines revealed significantly higher ALDH1A1 expression and activity in taxane- and platinum-resistant cell lines [31].

The ECM model of drug resistance development postulates that the small population of cells in the tumor is resistant to chemotherapy because of the high expression of ECM molecules and strong CAM-DR [10,33,34]. Usually, these cells survive chemotherapy and the number of ECM expressing cells increases after chemotherapy. It has been observed that ovarian cancer with a high level of COL3A1 expression was more resistant to chemotherapy [35]. ECM-mediated drug resistance seems to be one of the main reasons for platinum-sensitive recurrence in ovarian cancer [36].

In our study, the drug-resistant ovarian cancer cell lines revealed a strong expression of drug transporters [37,38], the presence of ALDH1A1+ population [19,29], and expression of many ECM molecules [20,21,39], especially collagens [22,40]. A more detailed co-expression study showed that drug-resistant cell lines present cells with different ECM expression levels, but the highest ECM expression is observed in ALDH1A1+ cells [19,22]. Based on our results, we created our drug resistance development model that combines CSCs and ECM models [19]. In this study, we used PAC- (W1PR1) and TOP-resistant (W1TR) ovarian cancer cell lines that we had previously developed from drug-sensitive W1 ovarian cancer cell line in the traditional 2D culture condition [38]. Using ALDEFLUOR assay and Western blot analysis, we identified two different populations of cells in W1TR and W1PR1 cell lines: a population lacking ALDH1A1 expression (ALDH1A1−) and a subpopulation with a high ALDH1A1 expression (ALDH1A1+) [19,29]. ALDH1A1+ cells were not present in the W1 sensitive cell line [19,29]. This study aimed to investigate the significance of ALDH1A1+ cells in the maintenance of drug resistance in ovarian cancer cell lines and the biological effects of *ALDH1A1* gene knockout.

## 2. Results

### 2.1. Model of the Study

In this study, we used drug-sensitive and resistant ovarian cancer cell lines. Previously, from a drug-sensitive W1 cell line, we derived cell lines resistant to six cytotoxic drugs, among them a W1PR1 cell line resistant to PAC and a W1TR cell line resistant to TOP [38]. In both cell lines ALDH1A1 positive CSCs were present in the content of 80% and 4%, respectively [29]. Here, we generated two ALDH1A− negative cell lines, W1PR1-C7 and W1TR-1p17, through gene knockout of W1PR1 and W1TR cell lines, respectively. Additionally, from a W1TR cell line, we derived a W1TR-2p17 cell line that was characterized by the expression of ALDH1A1 in all cells.

### 2.2. ALDH1A1 Gene Knockout Using the CRISPR-Cas9 System

**Figure 1:**
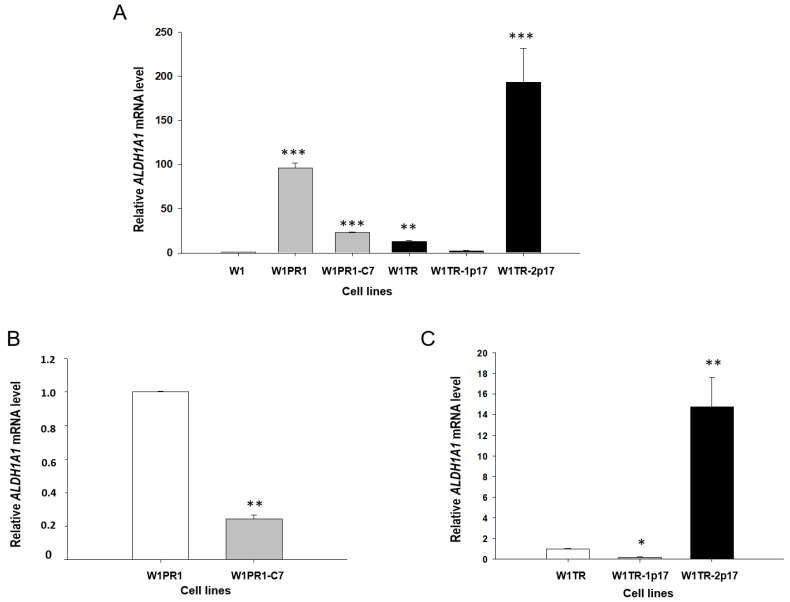
(**A**) Expression analysis of *ALDH1A1* transcript (Q-PCR) in the W1, W1PR1, W1PR1-C7, W1TR, W1TR-1p17 and W1TR-2p17 cell lines. The figure presents the relative gene expression in the PAC (paclitaxel)-resistant cell lines (gray bars), TOP (topotecan)-resistant cell lines (black bars) with respect to that in the sensitive cell line (W1) (white bar), which has been assigned a value of 1. (**B**) Expression analysis of *ALDH1A1* transcript (Q-PCR) in the W1PR1 and W1PR1-C7 cell lines. The figure presents the relative gene expression in W1PR1-C7 cell line (gray bar) with respect to that in the W1PR1 cell line (white bar), which has been assigned a value of 1. (**C**) Expression analysis of *ALDH1A1* transcript (Q-PCR) in the W1TR, W1TR-1p17, W1TR-2p17. The figure presents the relative gene expression in the W1TR-1p17 cell line (gray bar) and W1TR-2p17 cell line (black bar) with respect to that in W1TR cell line (white bar), which has been assigned a value of 1. The values were considered significant at * *p* < 0.05, ** *p* < 0.01, and *** *p* < 0.001.

To investigate the role of ALDH1A1 positive cells in the resistance to PAC and TOP, we generated a stable knockout of *ALDH1A1* gene in W1PR1 and W1TR cell lines by using the CRISPR-Cas9 system. Figure 1A presents a relative *ALDH1A1* gene expression in investigated cell lines. The transcript level in the W1 cell line was assigned as 1. We observed a statistically significant (*p* < 0.001) increase in *ALDH1A1* transcript level in both PAC resistant cell lines (W1PR1 and W1PR1-C7) in comparison to the W1 drug-sensitive cell line. However, the transcript level in W1PR1-C7 cell lines was about 5-fold lower than in the W1PR1 cell line (Figure 1A,B).

In the W1TR cell line, we observed an approximately 10-fold increase in *ALDH1A1* transcript level (*p* < 0.01) compared to the W1 cell line. The CRISPR-Cas9 mediated knockout of *ALDH1A1* gene in the W1TR-1p17 cell line resulted in downregulation of *ALDH1A1* mRNA level to the level similar to in the W1 cell line (Figure 1A). As we expected, the increase in *ALDH1A1* transcript level was observed in W1TR-2p17 cells, selected for ALDH1A1 positive cells. We noted about a 200-fold increase in comparison to W1 cell line (*p* < 0.001) (Figure 1A) and about a 15-fold increase in comparison to W1TR cell line (Figure 1C).

**Figure 2:**
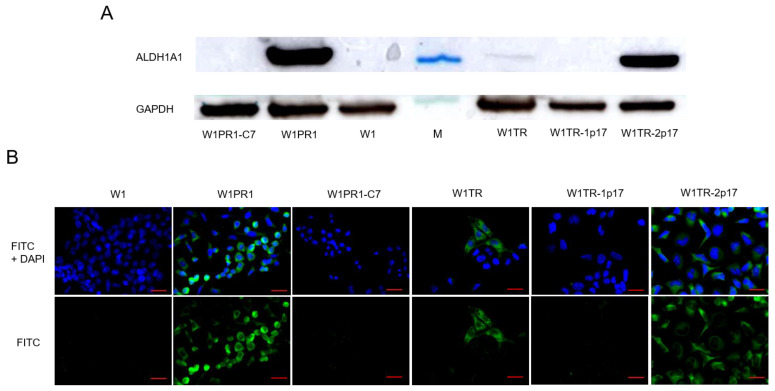
(**A**) ALDH1A1 protein expression analysis in the W1 drug-sensitive cell line and drug resistant W1PR1, W1PR1-C7, W1TR, W1TR-1p17, W1TR-2p17 cell lines. The cellular proteins were separated using 7% PAGE and transferred to a nitrocellulose membrane, immunoblotted with a primary antibody (Ab) and HRP-conjugated secondary Ab. A primary anti-GAPDH Ab served as a loading control for the cell lysates; M-protein molecular weight marker (**B**) Immunofluorescence visualization of ALDH1A1 expression in the W1 drug-sensitive cell line, drug resistant sublines and CRISPR-Cas9-edited variants. ALDH1A1 was detected using the anti-ALDH1A1 Ab and an Alexa Fluor^®^488-conjugated secondary Ab (green). DAPI was used to counterstain nuclei (blue). Objective 40×. Scale bar = 20 µm.

Knockout of the *ALDH1A1* gene was confirmed at the protein level by Western blot analysis (Figure 2A). The ALDH1A1 expression in drug-sensitive W1 and drug-resistant cell lines was assessed by Western blot analysis. W1 cells showed no ALDH1A1 protein expression. A very strong ALDH1A1 protein expression was observed in W1PR1 and W1TR-2p17 cell lines. Additionally, the presence of a weak band was observed in W1TR cell line.

To determine the expression of the ALDH1A1 protein in particular cells, we performed fluorescence analysis in investigated cell lines. We did not observe a fluorescence signal in the sensitive W1 cell line, consistent with our previous data [19,29]. We found that ALDH1A1 was selectively expressed in W1PR1 and W1TR cell lines resistant to PAC and TOP, respectively, also observed in our previous study [19,29] (Figure 2B). The knockout of *ALDH1A1* resulted in the lack of a fluorescence signal in all cells in W1PR1-C7 and W1TR-1p17 cell lines. In contrast, we observed a fluorescence signal in all cells from W1TR-2p17 cell line. Notably, the immunofluorescence results confirmed the Western blot data (Figure 2A,B).

### 2.3. MDR1/Glycoprotein- P, BCRP, COL3A1 Gene and Protein Expression in Ovarian Cancer Cell Lines

It appeared that PAC resistance in W1PR1 cell line was related to *MDR1*/P-gp overexpression [38] and resistance to TOP in W1TR cell line was related to BCRP [38] and COL3A1 [21,22] overexpression. Therefore, we were interested in whether *ALDH1A1* gene knockout would influence the expression of these drug-resistant proteins.

#### 2.3.1. *MDR1* Gene and Protein Expression in Investigated Cell Lines

**Figure 3:**
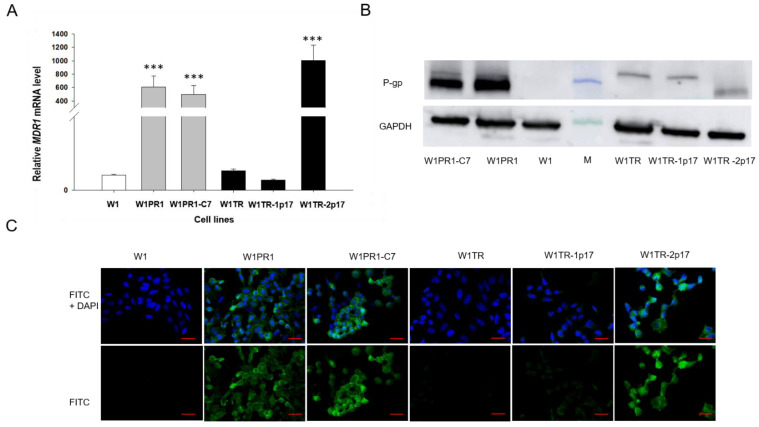
(**A**) Q-PCR analysis of *MDR1* transcript expression in the W1 cell line (white bar), W1PR1 and W1PR1-C7 cell line (gray bars), W1TR cell line, W1TR-1p17 and W1TR2-2p17 cell lines (black bars). The figure presents the relative gene expression in the resistant cell lines with respect to that in the sensitive W1 cell line, which has been assigned a value of 1. The values were considered significant at *** *p* < 0.001 (**B**) Western blot analysis of P-gp protein expression in the W1, W1PR1, W1PR1-C7, W1TR, W1TR-1p17 and W1TR-2p17 cell lines; M-protein molecular weight marker (**C**) Immunofluorescence visualization of P-gp expression in the W1, W1PR1, W1PR1-C7, W1TR, W1TR-1p17, W1TR2-2p17 cell lines. P-gp was detected using the anti-P-gp Ab and an Alexa Fluor^®^488-conjugated secondary Ab (green). DAPI was used to counterstain nuclei (blue). Objective 40×. Scale bar = 20 µm.

First, we were interested in whether the knockout of *ALDH1A1* gene affects *MDR1*/P-gp expression. Thus, the expression of *MDR1* transcript was determined by Q-PCR in all investigated cell lines. Our data seem to confirm our previous observation [38], as we noticed a 600-fold increment in *MDR1* mRNA level with statistical significance *p* < 0.001 in W1PR1 cell line (Figure 3A). Very similar *MDR1* expression was also observed in W1PR1-C7 cell line with no statistically significant difference between them (Figure 3A). The *MDR1* mRNA transcript level in W1TR and W1TR-1p17 cell lines was similar to W1 drug-sensitive cell line. In contrast, a substantial increase in *MDR1* mRNA level was observed in W1TR-2p17 cell line (*p* < 0.001). Next, we also compared protein expression in all cell lines. The elevated expression of P-gp was confirmed by Western blot analysis. We could observe a considerable increase in P-gp band intensity in both PAC-resistant W1PR1 and W1PR1-C7 cell lines compared to drug-sensitive W1 cell line and moderate band intensity in W1TR-2p17 cell lines (Figure 3B). The Western blot analysis provides a relative comparison of investigated protein levels in the whole cell population; however, the result may not correspond with the distribution of particular proteins among the entire cell population. To determine the expression of the P-gp protein in the investigated cell lines, we performed fluorescence analysis in parental cell line and resistant variants. We observed robust fluorescence signals in W1PR1, W1PR1-C7 and W1TR-2p17 cell lines (Figure 3C). Importantly, we observed uniform P-gp expression in cells from the same cell line. In contrast, we did not observe any fluorescence in drug sensitive W1 cell line as well as in W1TR and W1TR-1p17 cell lines (Figure 3C).

#### 2.3.2. *BCRP* Gene and Protein Expression in Drug-Resistant Ovarian Cancer Cell Lines

**Figure 4:**
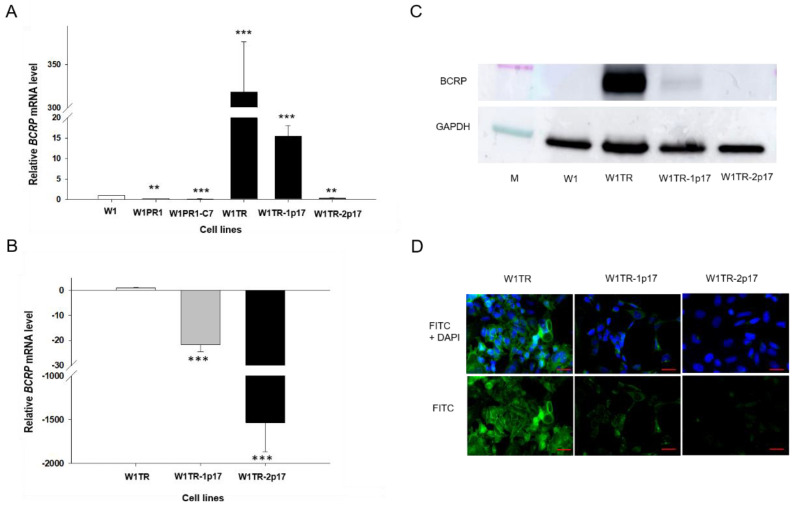
(**A**) Q-PCR analysis of *BCRP* transcript expression in the W1 cell line (white bar), W1PR1 and W1PR1-C7 cell line (gray bars), W1TR cell line, W1TR-1p17 and W1TR-2p17 cell lines (black bars). The figure presents the relative gene expression in the resistant cell lines with respect to that in the sensitive cell line, which has been assigned a value of 1; (**B**) Q-PCR analysis of *BCRP* transcript expression in the W1TR, W1TR-1p17 and W1TR-2p17 cell lines. The figure presents the relative gene expression in the CRISPR-Cas9-edited W1TR-1p17 resistant to TOP (topotecan) cell line (gray bar) and ALDH1A1+ W1TR-2p17 cell line (black bar) with respect to that in the resistant to TOP W1TR cell line, which has been assigned a value of 1 (white bar). The values were considered significant at ** *p* < 0.01 and *** *p* < 0.001. (**C**) Western blot analysis of BCRP protein expression in the W1, W1TR, W1TR-1p17 and W1TR-2p17 cell lines. A primary anti-GAPDH Ab served as a loading control for the cell lysates; M-protein molecular weight marker. (**D**) Immunofluorescence visualization of BCRP expression in W1TR, W1TR-1p17 and W1TR-2p17 cell lines. BCRP was detected using the anti-BCRP Ab and an Alexa Fluor^®^488-conjugated secondary Ab (green). DAPI was used to counterstain nuclei (blue). Objective 40×. Scale bar = 20 µm.

BCRP overexpression plays an essential role in resistance to TOP [5]. Previously, we observed increased expression of BCRP in all cells from the W1TR cell line [29]. Results obtained in this study are consistent with previous data as we observed a 300-fold increment (*p* < 0.001) in *BCRP* mRNA level in W1TR cell line when compared with W1 cell line (Figure 4A). Comparison of *BCRP* mRNA level between W1TR, W1TR-1p17 and W1TR-2p17 cell lines revealed statistically significant decrement (*p* < 0.001) in W1TR-1p17 and W1TR-2p17 cell lines when compared to W1TR cell line (Figure 4B). In W1TR-1p17 we observed about a 30-fold decrease in *BCRP* transcript compared to W1TR cell line (Figure 4B). A much stronger decrease in *BCRP* transcript was observed in W1TR-2p17 cell line (about 1500-fold) and was similar to the level observed in the drug-sensitive W1 cell line (Figure 4A,B). In W1PR1 and W1PR-1p17 cell lines, *BCRP* mRNA level was even lower than in W1 cell line. We confirmed the changes in expression of BCRP at the protein level by Western blot analysis. We observed a very strong BCRP band intensity in TOP-resistant W1TR cell line (Figure 4C). However, a weak band was observed in W1TR-1p17 cell line. Lack of BCRP band was observed in W1TR-2p17 cell line (Figure 4C) and drug-sensitive W1 cell line. Figure 4D shows BCRP protein expression confirmed at the cellular level in the immunofluorescence experiment. We detected a strong fluorescence signal in the drug-resistant W1TR cell line and low fluorescence signal in W1TR-1p17 cell line. Almost no detectible signal was observed in W1TR-2p17 drug sensitive cell line (Figure 4D).

#### 2.3.3. *COL3A1* Gene and Protein Expression in Drug-Resistant Ovarian Cancer Cell Lines

**Figure 5:**
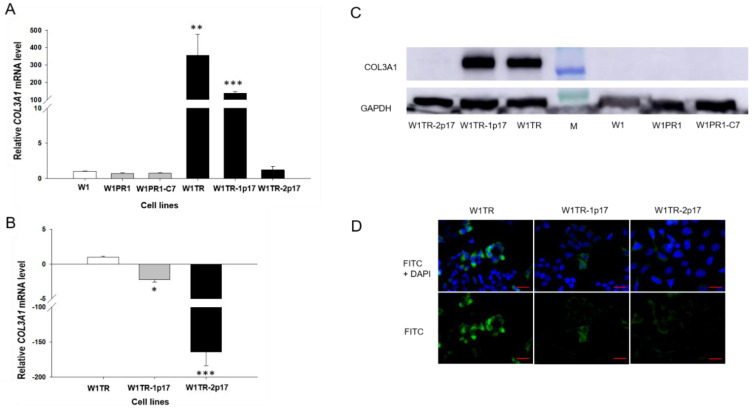
(**A**) Q-PCR analysis of *COL3A1* transcript expression in the W1, W1PR1, W1PR1-C7, W1TR, W1TR-1p17, W1TR-2p17 cell lines. The figure presents the relative gene expression in the resistant cell lines with respect to that in the sensitive cell line, which has been assigned a value of 1. (**B**) Q-PCR analysis of *COL3A1* transcript in the W1TR, W1TR-1p17 and W1TR-2p17 cell lines. The figure presents the relative gene expression in the CRISPR-Cas9-edited W1TR-1p17 resistant to TOP (topotecan) cell line (gray bar) and ALDH1A1+ W1TR-2p17 cell line (black bar) with respect to that in the resistant to TOP W1TR cell line, which has been assigned a value of 1 (white bar). The values were considered significant at * *p* < 0.05, ** *p* < 0.01, and *** *p* < 0.001. (**C**) Western blot analysis of COL3A1 protein expression in the W1, W1PR1, W1PR1-C7, W1TR, W1TR-1p17 and W1TR-2p17 cell lines. A primary anti-GAPDH Ab served as a loading control for the cell lysates; M-protein molecular weight marker. (**D**) Immunofluorescence visualization of COL3A1 expression in in W1TR, W1TR-1p17 and W1TR-2p17 cell lines. COL3A1 was detected using the anti-COL3A1 Ab and an Alexa Fluor^®^488-conjugated secondary Ab (green). DAPI was used to counterstain nuclei (blue). Objective 40×. Scale bar = 20 µm.

COL3A1 expression analysis was performed to estimate the association between *ALDH1A1* gene knockout and COL3A1 expression. Previously, we have demonstrated increased expression COL3A1 in ALDH1A1 positive cells [22]. Here, we observed a statistically significant increase of the *COL3A1* transcript in W1TR cell line (*p* < 0.01) and in W1TR-1p17 cell line (*p* < 0.001; Figure 5A). However, the expression of *COL3A1* was variable in these cell lines. We observed approximately 300-fold and 100-fold higher transcript levels in the W1TR and W1TR-1p17 cells, respectively, compared to drug-sensitive W1 cell line. Knockout of *ALDH1A1* gene resulted in about three-fold downregulation of COL3A1 transcript in W1TR-1p17 cell line (*p* < 0.05). However, in W1TR-2p17 cell line with overexpression of ALDH1A1, *COL3A1* transcript level decreased approximately 150-fold when compared to W1TR cell line (*p* < 0.001) (Figure 5B). The expression of COL3A1 at the protein level was confirmed by Western blot analysis. We observed very intense COL3A1 bands in both W1TR and W1TR-1p17 cell lines (Figure 5C). However, a lack of COL3A1 bands was observed in W1TR-2p17 cell line. In parental drug-sensitive cell line and PAC-resistant cell lines we also did not observe any COL3A1 band. The COL3A1 protein expression was confirmed at the cellular level in the immunofluorescence experiment. We noticed different fluorescence intensity dependently on the cell line. In W1TR cell line, two subpopulations of cells, one with high COL3A1 expression and another with moderate COL3A1 expression, were observed, consistent with our previous observations [22]. The W1TR-1p17 cell line also revealed two cell subpopulations concerning fluorescence intensity, but the general signal intensity was lower than in W1TR cell line. In W1TR-2p17, the fluorescence signal was almost detectable (Figure 5D).

### 2.4. The Morphological Characteristics of Cell Lines under 2D and 3D Cell Culture Conditions

**Figure 6:**
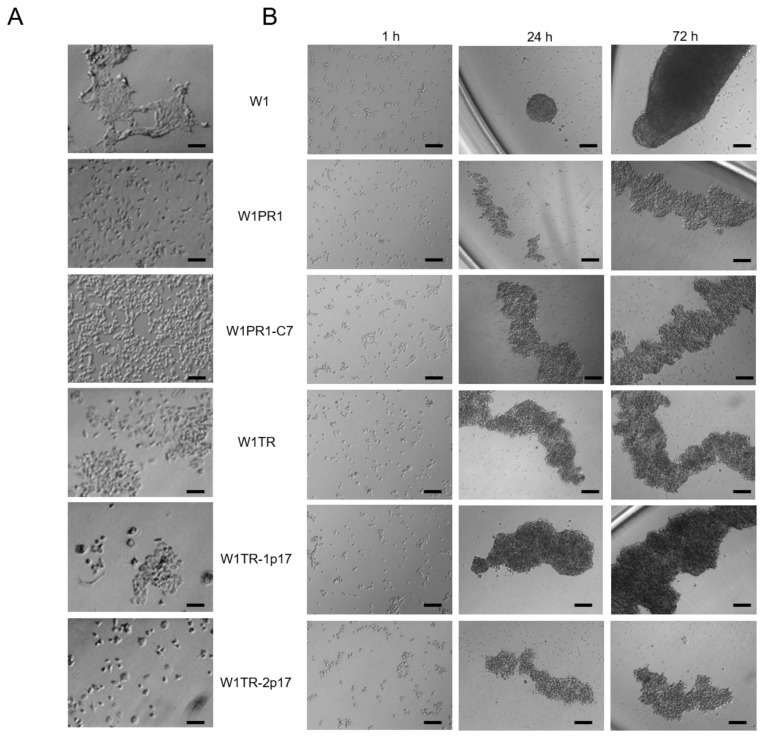
(**A**) Morphology of W1, W1PR1, W1PR1-C7, W1TR, W1TR-1p17 and W1TR-2p17 cell lines growing under 2D cell culture conditions. (**B**) Process of spheroid formation of W1, W1PR1, W1PR1-C7, W1TR, W1TR-1p17 and W1TR-2p17 cell lines. 10,000 cells of each cell line were seeded in 96-well plate. Images were acquired using an inverted microscope (Olympus). Objective 10×. Time in hours (h). Scale bar = 100 µm.

Because previously we had observed different morphology of drug-sensitive and resistant cell lines and various shapes of spheroids [41], we were interested in whether a knockout of *ALDH1A1* affects these parameters. In 2D conditions, W1, W1PR1, W1PR1-C7, W1TR, W1TR-1p17, W1 TR-2p17 cells grow as a monolayer in tissue culture flasks (Figure 6A). Parental cells show a fibroblastic-like phenotype with elongated cellular morphology and grow in cell clusters when W1PR1, W1PR1-C7, W1TR, W1TR-1p17, W1TR-2p17 cells exhibit more epithelial phenotype and grow as single cells (Figure 6A).

All examined cell lines formed spheroids (Figure 6B). Established spheroids differed in shape, density, and volume. W1PR1, W1PR1-C7, W1TR, W1TR-1p17, and W1 TR-2p17 cell lines formed elongated, cohesive aggregates while parental W1 cell line formed more sphere-like dense aggregates (Figure 6B).

### 2.5. Analysis of PAC and TOP Resistance of Cells Growing under 2D and 3D Cell Culture Conditions

#### 2.5.1. Analysis of PAC and TOP Resistance in 2D Conditions

**Figure 7:**
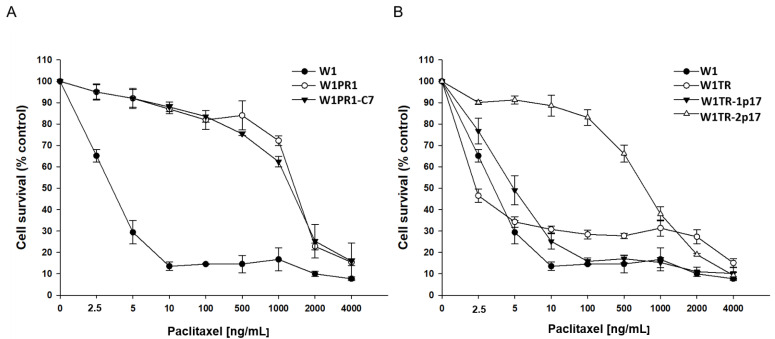
MTT cell survival assay in 2D cell culture conditions in W1, W1PR1 and W1PR1-C7 cell lines (**A**) and W1, W1TR, W1TR-1p17, W1TR-2p17 cell lines (**B**). Cells were seeded in 96-well plates and treated with or without increasing concentration of PAC (paclitaxel) at 37 °C for 72 h and viability of cells was determined. The experiments were repeated at least three times and each concentration was tested in triplicate in each experiment. Viability was expressed as a percentage on an untreated control (mean ± SEM).

We were interested in whether the level of drug resistance would reflect changes in ALDH1A1 and drug-resistance genes expression. All cell lines were treated with increased PAC concentrations for 72 h. After 72 h, cell survival assay was performed. Results for PAC- and TOP-resistant cell lines are presented in separate charts, Figure 7A,B, respectively. A drug-sensitive W1 cell line served as a control in both. As expected, we observed a reduction in cell viability in response to increased drug concentration. The comparison of IC50 between parental W1 cell line and resistant to PAC variants shows a significant increase of resistance to PAC in W1PR1 cell line (IC50 = 3.37 ng/mL vs. IC50 = 1671 ng/mL) and W1PR1-C7 cell line (IC50 = 3.37 ng/mL vs. IC50 = 1342 ng/mL). A minimal decrease in PAC resistance was observed in the *ALDH1A1*-knockout cell line in comparison to W1PR1 cell line (IC50 = 1671 ng/mL vs. IC50 = 1342 ng/mL) (Table 1).

Because in W1TR-2p17 cell line we detected expression of P-gp, we also compared resistance to PAC in TOP-resistant cell lines (Figure 7B). We observed a dose-dependent pattern of cell viability reduction for all cell lines, but for W1TR-2p17 cell line we detected the reduction of cell viability only at a high drug concentration (Figure 7B, Table 1). The comparison of IC50 between parental W1 cell line and cell lines resistant to TOP shows a minimal increase in PAC-resistance in W1TR-1p17 cell line (IC50 = 3.37 ng/mL vs. IC50 = 5.53 ng/mL) and strong increase in PAC resistance in W1TR-2p17 cell line (IC50 = 3.37 ng/mL vs. IC50 = 787 ng/mL). We also observed statistically significant differences in PAC resistance between W1TR and W1TR-2p17 cell lines (IC50 = 3.37 ng/mL vs. IC50 = 787 ng/mL) (Table 1).

**Figure 8:**
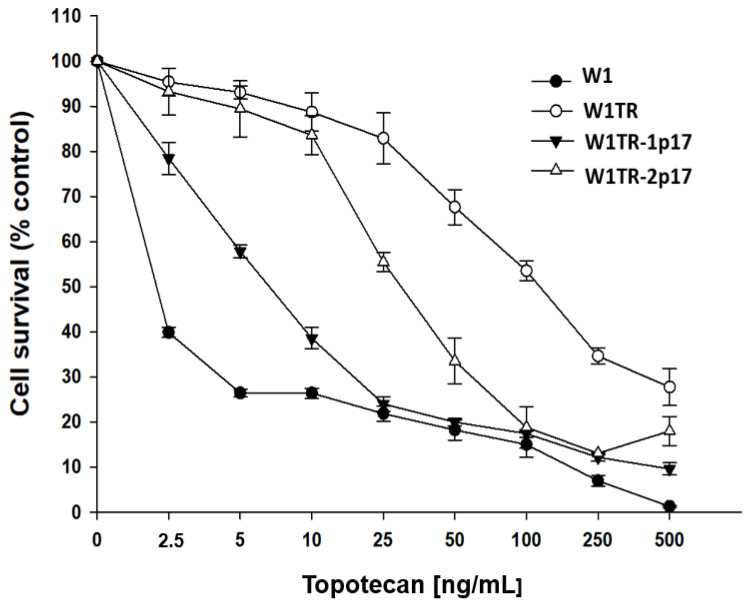
MTT cell survival assay in 2D cell culture conditions in W1, W1TR, W1TR-1p17 and W1TR-2p17 cell lines. Cells were seeded in 96-well plates and treated with or without increasing concentration of TOP (topotecan) at 37 °C for 72 h and viability of cells was determined. The experiments were repeated at least three times and each concentration was tested in triplicate in each experiment. Viability was expressed as a percentage on an untreated control (mean ± SEM).

Next, we compared TOP resistance level in W1, W1TR, W1TR-1p17 and W1TR-2p17 cell lines. All cell lines presented a dose-dependent reduction of cell viability in response to increased drug concentration (Figure 8). The comparison of IC50 between parental W1 cell line and TOP-resistant cell lines presented a strong increase in TOP resistance in W1TR cell line (IC50 = 2.06 ng/mL vs. IC50 = 111 ng/mL), a medium increase in W1TR-2p17 cell line (IC50 = 2.06 ng/mL vs. IC50 = 31.1ng/mL) and a low increase in TOP-resistance in W1TR-1p17 cell line (IC50 = 2.06 ng/mL vs. IC50 = 7.18 ng/mL) (Table 1). In both cell lines derived from W1TR cell line, we observed a reduction in TOP-resistance compared to W1TR cell line (IC50 = 111 ng/mL vs. IC50 = 7.18 ng/mL or IC50 = 31.1 ng/mL).

#### 2.5.2. Analysis of PAC and TOP Resistance of Cells Growing under 3D Cell Culture Conditions

**Figure 9:**
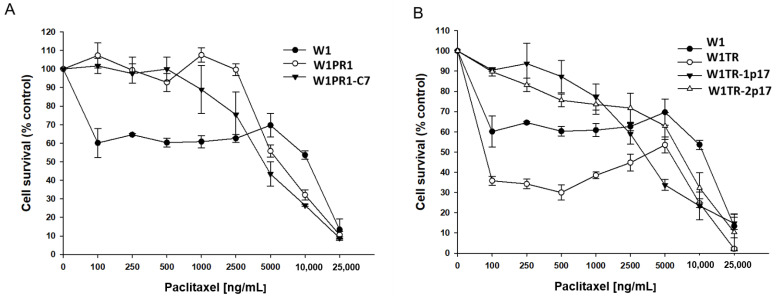
MTT cell survival assay in 3D cell culture conditions in W1, W1PR1 and W1PR1-C7 cell lines (**A**) and W1, W1TR, W1TR-1p17, W1TR-2p17 cell lines (**B**). Cells were seeded in 96-well plates with non-adherent surface and treated with or without increasing concentration of PAC (paclitaxel) at 37 °C for 72 h and viability of cells was determined. The experiments were repeated at least three times and each concentration was tested in triplicate in each experiment. Viability was expressed as a percentage on an untreated control (mean ± SEM).

Next, we investigated the response to PAC and TOP of the same cell lines growing as spheroids. After spheroids were formed, cells were incubated with increased PAC concentration for 72 h. The results for PAC- and TOP-resistant cell lines are presented in separated charts. (Figure 9A,B, respectively). The drug-sensitive W1 cell line was used as a control for both. For W1 cell line, we observed a three-step response curve. Lower PAC concentration (100 ng/mL) reduces cell viability to about 60% of control. Further increase of PAC concentration up to 5000 ng/mL did not influence cell viability. Only very high PAC concentrations were able to reduce spheroids’ viability (Figure 9A). In the case of W1PR1 and W1PR1-C7 cell lines, we observed a very similar response pattern, and only a very high drug concentrations were able to decrease cell viability (Figure 9A). Table 2 summarizes the IC50 value for investigated cell lines. Comparison of IC50 between W1 and W1PR1 and W1PR1-C7 cell lines showed that in spheroids W1 cell line was more resistant to PAC than W1PR1 and W1PR1-C7 cell lines (IC50 = 11,883 ng/mL vs. 6022 ng/mL or 3971 ng/mL, respectively). We also observed a reduction in spheroids viability in ALDH1A1 knockout cell line compared to W1PR1 cell line (6022 ng/mL vs. 3971 ng/mL), although not statistically significant (Table 2).

A three-step response curve was also observed in W1TR cell line (Figure 9B). Initially, W1TR cells treated with low PAC concentrations (100–500 ng/mL) exhibited poor survival (only about 35% compared to controls). Another increase in drug concentration up to 5000 ng/mL resulted in a moderate increase in cell survival, and only a very high drug concentration resulted in cell death (Figure 9B). More linear decrement in viability was observed for W1TR-1p17 and W1TR-2p17 cell lines (Figure 9B). Comparison of IC50 value in spheroids from W1, W1TR, W1TR-1p17 and W1TR-2p17 shows that W1 spheroids are more resistant to PAC than spheroids from W1TR, W1TR-1p17 and W1TR-2p17 cell lines (IC50 = 11,883 ng/mL vs. IC50 = 6099 ng/mL or IC50 = 3283 ng/mL and IC50 = 7183 ng/mL). Comparison of IC50 value of W1TR cell line and W1TR-1p17 revealed statistically significant decrease in resistance in W1TR-1p17 cell line (IC50 = 6099 ng/mL vs. IC50 = 3283 ng/mL) (Table 2).

**Figure 10:**
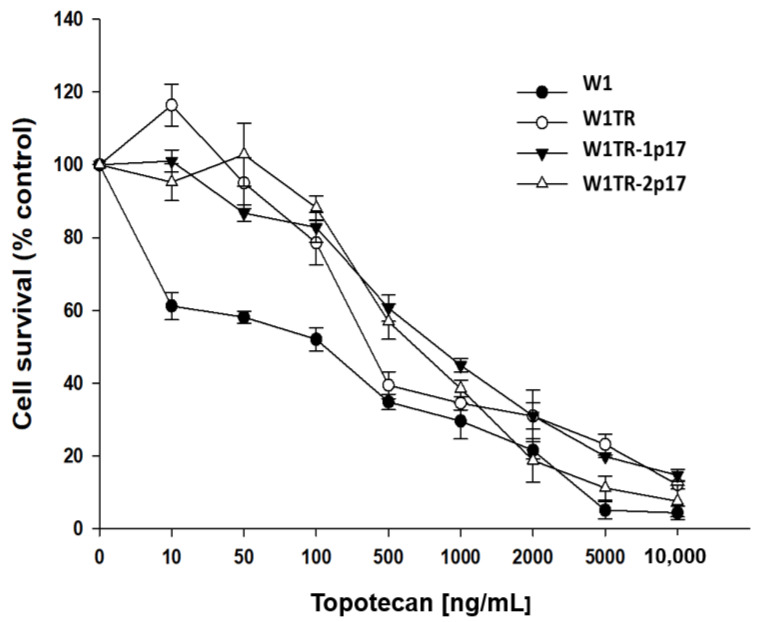
MTT cell survival assay in 3D cell culture conditions in W1, W1TR, W1TR-1p17 and W1TR-2p17 cell lines. Cells were seeded in 96-well plates with non-adherent surface and treated with or without increasing concentration of TOP (topotecan) at 37 °C for 72 h and viability of cells was determined. The experiments were repeated at least three times and each concentration was tested in triplicate in each experiment. Viability was expressed as a percentage on an untreated control (mean ± SEM).

We observed a concentration-dependent response to TOP in all examined cell lines (Figure 10). A comparison of IC50 values in investigated spheroids revealed that spheroids derived from W1 cell line were more sensitive to TOP than spheroids derived from W1TR, W1TR-1p17 and W1TR-2p17 cell lines (IC50 = 154 ng/mL vs. IC50 = 384 ng/mL vs. IV50 = 814 ng/mL and IC50 = 697 ng/mL) (Table 2). Because W1TR-1p17 and W1TR-2p17 cell lines were derived from W1TR cell line, we also compared the resistance of these spheroids. Direct comparison of spheroids from W1TR, W1TR-1p17 and W1TR-2p17 cell lines shows further increase in spheroids’ resistance (IC50 = 384 ng/mL vs. IC50 = 814 ng/mL and IC50 = 697 ng/mL) (Table 2).

## 3. Discussion

Drug resistance is one of the main problems in the treatment of cancers. While some cancers are intrinsically resistant to chemotherapy, others develop resistance in the course of drug treatment [42]. Below 20% of ovarian cancer cases are primarily resistant to chemotherapy, and about 80% of primary sensitive patients develop drug resistance during treatment [43]. According to the CSCs model of drug resistance development, a small population of cells is characterized by resistance to chemotherapy survive treatment and is responsible for the recurrence of drug resistance tumors [26,44,45]. ALDH1A1 is a universal marker of CSCs among solid tumors, including OC (Ovarian Cancer) [31,46]. Various studies have reported that high ALDH1A1 expression correlates with decreased overall survival (OS) in OC patients [47,48,49], reduced progression-free survival (PFS) [50], lower response to chemotherapy [50], and an increased number of ALDH1A1+ cells observed in ovarian tumors after CIS/PAC combined chemotherapy [51]. The presence of ALDH1A1+ cells was observed both in vivo in ovarian cancer patients and in vitro in drug-resistant OC cell lines. It has been observed that ALDH1A1+ cells isolated from OC cell lines were more resistant to chemotherapy [48,49]. However, the number of ALDH1A1+ cells differs significantly among drug-resistant cell lines. In the paper of Laden et al. [31], the presence was observed of a small percentage of ALDH1A1+ cells in A2780 CIS-resistant cell line and about 50% in SKOV-3 PAC-resistant cell line. In another study, authors observed about 40% of ALDH1A1+ cells in A2780-PAC resistant cell line [52]. In our study [29], we observed nearly 80% of ALDH1A1+ cells in W1PR1 cell line (PAC-resistant), and most of these showed high ALDH1A1 expression and activity. Eventually, in TOP-resistant cell line (W1TR), we observed only 4% of cells with ALDH1A1 activity [29].

Although the presence of a cytotoxic drug in the environment induces chemoresistance in TOP- and PAC- resistant variants of W1 cell line, not all cells express ALDH1A1. This suggests that ALDH1A1 expression is not a leading component of the TOP- and PAC-resistance phenomenon. Evidence suggests that ALDH1A1 expression is directly involved in resistance to cyclophosphamide [53,54] but any available data do not show a direct contribution of ALDH1A1 to TOP- and PAC- resistance.

According to the CSCs model of drug resistance development, CSCs are intrinsically resistant to chemotherapy because of the expression of drug transporters such as P-gp or BCRP and the presence of another resistance mechanism [23,45,55]. After chemotherapy, only CSCs survive and rebuild the tumor mass [26,44,45]. Recently, we observed increased expression of ECM molecules, especially collagens in ALDH1A1+ cells suggesting an important role of CAM-DR in the resistance of CSCs [19,22]. However, CSCs can divide asymmetrically, giving a CSC and more differentiated cells which, under the cytotoxic environment, remain more resistant [19,45]. The question is whether or not the expression of ALDH1A1+ cells is required for the maintenance of drug resistance for a long time. Knockout of *ALDH1A1* gene in W1PR1 (80% of ALDH+ cells) and W1TR (4% of ALDH+ cells) cells resulted in a total loss of ALDH1A1 expression and lack of ALDH1A1+ cells. However, knocking out the *ALDH1A1* gene did not influence *MDR1*/P-gp expression, suggesting that ALDH1A1 expression is not required for *MDR1* gene expression in this cell line. Different ALDH1A1 dependent and independent regulation of drug transporters’ expression has been described in the literature [27,56,57]. In a study by Hafiz Uddin et al. in A2780, CIS-resistant cell line expression of *MDR1*/P-gp, BCRP and MRP1 was regulated by ALDH1A1-NEK-2 axis, and silencing of ALDH1A1 expression resulted in downregulation of P-gp, MRP1, and BCRP expression and increased sensitivity to CIS [27]. Very similar ABCB1 regulation by ALDH1A1-NEK-2 axis was also observed in multiple myeloma [56], suggesting the significance of high ALDH1A1 regulating ABC transporters’ expression. Another ALDH1A1-dependent pathway of drug transporters regulation has been described in the ALDH1A1+ population isolated from the A2780-DOX resistant OC cell line [57]. Two cell subpopulations: ALDH1A1-low(L) and ALDH1A1-high(H), presented P-gp and BCRP proteins expression with higher levels for the ALDH1A1(H) population, which resulted in some increase in PAC and DOX resistance of these cells. Silencing ALDH1A1 expression by siRNA or ATRA treatment resulted in decreased drug transporters’ expression [57]. P-gp and BCRP expression in ALDH1A1 (L) population were independent of ATRA (All-trans retinoic acid) treatment, suggesting a different regulation mechanism for those proteins between ALDH1A1 (H) and ALDH1A1 (L) cells’ population [57]. In our experiment, ALDH1A1- cell line revealed high P-gp expression, hence our model’s regulation of P-gp seems to be ALDH1A1 independent.

Resistance to TOP in the W1TR cell line was related to the uniform expression of BCRP protein in all cells. Additionally, in this cell line, we previously observed cells with very high expression of COL3A1, which was secreted to the cell culture medium forming a spider web structure, therefore also suggesting the role of COL3A1 in TOP resistance [19,22]. Furthermore, COL3A1 was expressed mainly by ALDH1A1+ cells [19]. Knockout of *ALDH1A1* in this cell line resulted in complete loss of ALDH1A1 protein expression and lack of ALDH1A1+ cells. However, in contrast to the W1PR1 cell line, we observed uniform decreased expression of BCRP protein, suggesting the role of ALDH1A1 in regulating BCRP expression. The role of ALDH1A1 pathways in the regulation of BCRP expression has been described [27,57]. However, this can be true only for a small population of ALDH1A1+ cells. The reason for decreased BCRP in ALDH1A1- cells is difficult to explain. *ALDH1A1* gene knockout also resulted in reduced expression of COL3A1, but this was, rather, related to a decrease in the number of ALDH1A1/COL3A1+ cells. The regulation of COL3A1 expression by ALDH1A1 has never been described so far.

Surprisingly, in the W1TR-2p17 cell line, characterized by ALDH1A1 expression in all cells, we observed loss of BCRP and COL3A1 expression related to TOP-resistance in the parental W1TR cell line [22,38]. This should sensitize these cells, but the loss of BCRP and COL3A1 expression seems to be compensated for by the high expression of *MDR1*/P-gp in this cell line.

We observed differences in 2D morphology as well as density and shape of 3D spheroids between W1 and drug-resistant cell lines. However, changes in ALDH1A1 expression did not change cell morphology and spheroids’ shape. It suggests that the determinant of cells morphology and the features of spheroids is a drug-sensitive/resistant phenotype but not the presence of ALDH1A1+ cells.

After knockout experiments we performed a cytotoxicity assay to determine how *ALDH1A1* gene knockout influences the chemosensitivity of cells. In 2D cell culture conditions, all cells are equally exposed to cytotoxic agents, and the level of resistance should be related to the expression of drug resistance proteins. Contrary to our expectations, W1PR1 and W1PR1-C7 cells exhibited similar chemoresistance levels, suggesting that *ALDH1A*1 expression is not a key player in PAC-resistance. The PAC-resistance of those cells seems instead to be driven by abundant P-gp expression, as PAC is a well-known substrate of P-gp [5,6,7]. Furthermore, W1TR-2p17 cells demonstrated a higher PAC-resistant phenotype than we expected. This phenomenon seems to be related to the level of *MDR1*/P-gp expression. This further confirms that the primary role in PAC-resistance at the cellular level is an expression of P-gp. Moreover, BCRP was described as a prominent protein in TOP-resistance [9], and our previous research is consistent with literature data [37,58,59]. In the next step, we evaluated the impact of *ALDH1A1* gene knockout on BCRP expression and TOP-resistance.

Knockout of *ALDH1A1* gene in TOP-resistant cell line resulted in decreased TOP-resistance and correlated with a decrease in BCRP protein level. The above observation confirms the role of BCRP protein in TOP-resistance, and provides more insight into the mechanism of cells’ resensitization regulated by ALDH1A1 inhibition that we described previously [29].

In a recent study, we have proposed the role of COL3A1 expression in TOP-resistance [22]. In this study, we observed abundant expression of COL3A1 in TOP-resistant W1TR and W1TR-1p17, but the cell line with knocked out *ALDH1A1* gene showed lower expression of the *COL3A1* gene and protein when compared with parental TOP-resistant cell line. To our best knowledge, the decrement of TOP-resistance in W1TR-1p17 cell line might be linked with decreased COL3A1 expression. However, further studies are required on the precise mechanism of this dependence. Although the expression of BCRP and COL3A1 in W1TR-2p17 cell line was low, we observed a medium level of TOP-resistance, which suggests other forms of mechanism-driven resistance. Based on literature data and our knowledge, we know that TOP-resistance can also be related to P-gp expression [42,58,59,60,61]. P-gp dependent resistance to TOP was observed in W1PR1 cell line [38]. Thus, the resistance of W1TR-2p17 cell line to TOP seems to result from a high level of P-gp expression.

The traditional 2D cell culture method is a well-known and relatively simple cytotoxicity assessment method. However, it does not reflect tumor tissue conditions where interaction between tumor cells and tumor microenvironment can influence drug response [62]. Therefore, we also applied 3D cell culture technics to better reflect the cancer tissue environment. In contrast to a 2D cell culture condition in spheroids, we observed a different response to PAC and TOP treatment.

Surprisingly, in 3D cell culture conditions W1 cell line was more resistant to PAC than the drug resistant W1PR1 cell line and W1PR1-C7 variant. Additionally, W1TR cells were far less resistant to TOP than W1 cells when growing as spheroids, and not as a monolayer (2.5-fold in 3D vs. 50-fold in 2D). In W1 spheroids treated with either PAC or TOP, we did not observe a typical dose-dependent response as in the 2D condition, but rather a three-step response curve. Initially, low PAC concentration decreases cell viability significantly, but further increasing the dose of PAC did not enhance the response up to extremely high PAC concentration, resulting in a cytotoxic effect. In contrast to W1 cell line, W1PR1 and W1PR1-C7 cell lines were resistant to lower PAC-concentration, and only high drug concentration was able to reduce spheroids’ viability. A similar observation was previously made by our team for spheroids derived from A2780 PAC-resistant cell lines [41]. The possible explanation is a dense cellular structure and the presence of ECM molecules in W1 that can limit drug diffusion into the central part of the spheroid [63]. Moreover, it has been reported that PAC can bind to cellular macromolecules which limit its availability to cancer cells [64]. The second reason is that a spheroid is composed of different zones. At the periphery highly proliferating cells are present, in an intermediate zone viable and congenic but static cells are present, and in an inner core necrotic cells can be found [63]. As cytotoxic drugs, especially PAC, were designated to target highly proliferating cells [65], low PAC concentration cannot kill static/slowly dividing cells inside the spheroid. Only high PAC concentration can reduce spheroid viability. Different response curves from PAC-resistant cell lines result from the high expression level of P-gp. Cells from the proliferative zone are not sensitive to low PAC concentration because of high P-gp expression. Increased PAC concentration above the efficiency of P-gp can kill cells from this zone, and then reduce the viability of the whole spheroid.

In W1 spheroids treated with TOP, low drug dose reduced spheroids’ viability to about 60% via action on highly proliferating cells from the peripheral zone. This can be explained by TOP’s ability to inhibit topoisomerase I which is active in highly proliferating cells [66,67]. A further 10-fold increase in TOP concentration did not significantly change spheroids’ viability, and only higher TOP-concentration reduced this. A similar response pattern was observed in spheroids of W1TR, W1TR-1p17, and W1TR-2p17 cell lines.

It has also been shown that some ECM proteins can inhibit TOP-induced apoptosis in a dose-dependent manner [68]. The other role of COL3A1 can also result from limited drug diffusion by the collagen network and the possible binding of TOP to COL3A1 molecules. According to literature data, extracellular collagen can limit drug diffusion into tumors [12,69]. Increased expression of COL3A1 has been reported in CIS-resistant ovarian tumors [35]. It is also highly possible that a very high expression level of COL3A1 in W1TR and W1TR-1p17 cell lines results in TOP binding. It has been reported that drugs like CIS or 5-fluorouracil easily migrate within the tumor [70]. In contrast, vinblastine (VIN), DOX, or PAC bind to cellular macromolecules that limit their accessibility to tumor cells [64].

Summarizing this part of the investigation, we can conclude that drug response in a 2D cell culture condition is related to the expression level of drug resistance molecules and, in our study, mainly P-gp and BCRP. Reaction to drug in 3D spheroids is related to their properties, such as spheroid density and expression of ECM molecules, expression of drug transporters and ability of a drug to migrate into the spheroid. In general, in all cell lines, we observed an increase in drug resistance in 3D condition compared to 2D conditions. An extreme increase in resistance to both PAC and TOP was observed in drug-sensitive W1 cell lines that correspond to high cell density in this spheroid. Our results are in line with others, showing higher resistance of 3D spheroids than 2D cell culture, and thus 3D cell culture methods appear adequate in simulating important tumor characteristics [71,72,73]. However, there is a potential limitation in this study that could be addressed in future research. The study focused on the impact of *ALDH1A1* gene knockout on drug resistance in a model of drug sensitive and drug resistant cell lines. Further studies are needed to evaluate the contribution of *MDR1*/P-gp and BCRP in this system.

## 4. Materials and Method

### 4.1. Cell Lines and Cell Culture

The human primary ovarian cancer cell line W1 was established from the tumor tissue of an untreated 54-year-old Caucasian female patient diagnosed for serous ovarian adenocarcinoma (G3. FIGO IIIc) [20]. Cells grow as a monolayer and present an epithelial morphology and adherent growth model. The W1 subline, resistant to PAC (W1PR1) and resistant to TOP W1TR, were generated by exposing parental cells to PAC or TOP, respectively, at increasing concentrations. The final concentrations used for selecting the resistant cells were 1100 ng/mL of PAC and 24 ng/mL of TOP. In W1PR1 we observed ALDH1A1 activity in about 80% of cells and in W1TR cell line we observed ALDH1A1 activity in about 4% of cells [29]. All the cell lines were maintained as monolayers in complete growth medium RPMI supplemented with 10% (*v*/*v*) fetal bovine serum (FBS), 2 pML-glutamine, penicillin (100 units/mL), streptomycin (100 units/mL) and amphotericin B (25 μg/mL) at 37 °C in a 5% CO_2_ atmosphere. Culture medium (RPMI-1640), fetal bovine serum, antibiotic-antimycotic solution, L-glutamine, BSA, DAPI mounting medium, TOP and PAC were purchased from Sigma (St. Louis, MO, USA). To create 3D spheroids, cells were trypsinized, resuspended in the growth medium and transferred to a nonadherent surface 96-wells plate (BRAND, Germany, plates inertGrade, F-bottom) in 10,000 cells per well. Phase images of 2D and 3D cell culture were acquired using an inverted microscope (Olympus IX73, Tokyo, Japan).

### 4.2. Stable Transfection with ALDH1A1 Gene Knockout Plasmids

The *ALDH1A1* gene editing was performed in W1PR1 and W1TR cells using CRISPR-Cas9 gene-editing technology. The HDR (Homology Directed Repair) mediated CRISPR/Cas9 knockout kit was used in this study (OriGene Technologies, Rockville, MD, USA, catalog No. KN200723). The CRISPR/Cas9 knockout system used in this study consists of 3 plasmids: *ALDH1A1* gRNA vector 1 Target Sequence: GAATCTTCAAATCGGTGAGT (KN200723G1), ALDH1A1 gRNA vector 2 Target Sequence: AGGTAAGTCTGGCGTGCCTG (KN200723G2), donor DNA containing left and right homologous arms and GFP-puro functional cassette (KN200723-D). The plasmids were transfected into the W1PR1 and W1TR cells by electroporation (Neon Transfection System, Invitrogen by Thermo Fisher Scientific, Waltham, MA, USA) according to manufacturer’s protocol. After the indicated selection period with puromycine, single-cell colonies were picked and analyzed for ALDH1A1 expression at the mRNA and protein levels. As a result, we obtained W1PR1 cell line with *ALDH1A1* gene knockout. This cell line was named W1PR1-C7. We also obtained W1TR cell line with *ALDH1A1* gene knockout and this cell line was named W1TR-1p17. In parallel with the W1TR cell line we also developed by clonal selection a subline with expression of ALDH1A1 in all cells to create a similar condition to *ALDH1A1* knockout cell lines. This cell line was also transfected with donor DNA containing left and right homologous arms and GFP-puro functional cassette (KN200723-D) and was cultured in the presence of puromycin.

### 4.3. MTT Assay

The drug resistance of the investigated cell lines was determined by the MTT cell survival assay (Cell Proliferation Kit, Roche, GmbH, Mannheim, Germany). For 2D culture 4000 cells for 3D culture 10,000 cells of each cell line were seeded in each well of the 96-well plates. After 48 h of culturing (2D and 3D) cells were treated with fresh medium supplemented with or without increasing concentrations of PAC and TOP. After 72 h of incubation at 37 °C, the MTT experiments were performed according to the manufacturer’s protocol. The absorbance was measured using a microplate reader (BioTek (Agilent), Santa Clara, CA, USA) at 570 nm with a reference wavelength of 720 nm, according to the manufacturer’s protocol. As a negative control, a cell-free culture medium containing the MTT reagents was used. Each experiment was repeated three times and each concentration in a given experiment was tested in duplicate. The IC50 value of both drugs was determined in each cell line.

### 4.4. Examination of Gene Expression by Q-PCR

We analyzed *ALDH1A1, MDR1, BCRP,* and *COL3A1* gene expression in examined cell lines. According to the protocol, RNA isolation was performed with the Gene Matrix Universal RNA Purification Kit (EURx, Ltd., Gdansk, Poland), and Q-PCR experiments were performed using 2 μg of RNA for cDNA synthesis and the M-MLV reverse transcriptase kit (Invitrogen by Thermo Fisher, Waltham, MA, USA) and thermal cycler (T100, Bio-Rad Laboratories, Hemel Hempstead, UK). Table 3 presents primers used in the study. Real-time PCR analysis was performed using a 7900 HT Fast Real-Time PCR System (Applied Biosystems, Foster City, CA, USA). The geometric mean of the following reference genes was calculated: glyceraldehyde 3-phosphate dehydrogenase (*GAPDH*), β-actin, hypoxanthine-guanine phosphoribosyl-transferase 1 (*HRPT1*) and beta-2-microglobulin (*β2M*). The graphs were generated using SigmaPlot 11.0 (Systat Software, Erkrath, Germany). The amplification was performed with the use of 12.5 μL of ROX SYBR qPCR Master Mix (Takyon, Eurogentec, Angers, France), 1 μL of each primer (Oligo, Warsaw, Poland, Table 3), 9.5 μL of MQ water and 1 μL of cDNA solution. For each experiment, one RNA sample was processed without the RT-reaction (negative control). The process of amplification included a hot start (95 °C, 15 min), 45 cycles of denaturation (95 °C for 15 s), annealing (60 °C for 30 s), and extension (72 °C for 30 s). After amplification, melting curves were used to determine the specificity of the gene products.

### 4.5. Western Blot Anlysis

Protein levels were assessed by Western blotting. Cell lysates were prepared using a RIPA buffer (Sigma-Aldrich, Poznan, Poland) with a protease inhibitor cocktail (Roche Diagnostics GmbH, Mannheim, Germany). After centrifugation (4 °C and 12.000× *g* for 10 min) supernatant was collected and the Bradford protein assay system (Bio-Rad Laboratories, Hemel Hempstead, UK) was applied to determine the protein concentrations. For the Western blot analysis, proteins (20 μg from each sample and loading buffer- Bio-Rad Laboratories, Hemel Hempstead, UK) and protein molecular weight marker (Bio-Rad Laboratories, Hemel Hempstead, UK) were separated via 4–20% mini-PROTEAN^®^-TGX precast gel using the SDS-PAGE technique and afterward transferred to a nitrocellulose membrane (Bio-Rad Laboratories, Hemel Hempstead, UK). In the next step, the membrane was incubated in blocking buffer (5% milk in TBS/Tween20 (0.1 M Tris-HCl. 0.15 M NaCl 0.1% Tween 20)) for 1 h. Next, the membrane was incubated overnight with an appropriate primary antibody (Ab) followed by incubation with the corresponding HRP-conjugated secondary Ab. The following primary antibodies and dilutions were used: rabbit monoclonal anti-ALDH1A1 at dilution of 1:200 (Abcam, Cambridge, UK), mouse monoclonal anti-P-gp ab used at dilution 1:50 (Invitrogen, Thermo Fisher Scientific, Waltham, MA, USA), rabbit monoclonal anti-BCRP Ab at dilution 1:1000 (Cell Signaling, Danvers, MA, USA), mouse polyclonal ant- COL3A1 ab at dilution 1:1000 (Proteintech, Manchester, UK), rabbit polyclonal anti-GAPDH Ab at dilution 1:1000 (Santa Cruz Biotechnology, Santa Cruz, CA, USA). The following secondary antibodies and dilutions were used: anti-rabbit HRP-conjugated Ab, 1:1000 (Cell Signaling, Danvers, MA, USA) and anti-mouse HRP-conjugated Ab. 1:1000 (Cell Signaling, Danvers, MA, USA). The bands’ detection was analyzed using a chemiluminescence detection kit (Bio-Rad Laboratories, Hemel Hempstead, UK) and Hyperfilm ECL (GE Healthcare, Buckinghamshire, UK).

### 4.6. Immunofluorescence Analysis

For immunofluorescence analysis, cells were cultured in 24-well chamber glass slides. Cells were washed with PBS and next incubated in ice-cold acetone/methanol (1:1) for 10 min for fixation and permeabilization. Then, cells were washed with PBS and blocked in 3% BSA (Bovine Serum Albumin) for 30 min at room temperature. After that, cells were incubated with relevant primary antibodies. The following primary antibodies and dilutions were used: rabbit monoclonal anti-ALDH1A1 at dilution 1:100 (Abcam, Cambridge, UK), mouse monoclonal anti-P-gp at dilution 1:50 (Invitrogen, Thermo Fisher Scientific, Waltham, MA, USA), rabbit monoclonal anti-BCRP at dilution 1:300 (Cell Signaling, Danvers, MA, USA), mouse polyclonal ant- COL3A1 at dilution 1:100 (Proteintech, Manchester, UK). After incubation with primary antibodies (2 h, room temperature), cells were washed with PBS and incubated with corresponding secondary antibodies (green dye-labeled Alexa Fluor 488 secondary Ab: Donkey Anti-Mouse IgG and Donkey Anti-Rabbit IgG both purchased from Jackson Immuno Research Laboratories, Cambridgeshire, UK) for 1 h at room temperature. Finally, cells were washed with PBS and mounted in DAPI mounting medium. The expression analysis and pictures were taken with a fluorescence microscope (Zeiss Axio-Imager.Z1, Munich, Germany).

### 4.7. Statistical Analysis

The statistical analysis was performed using Excel 2016 software (Microsoft, Redmont, WA, USA). The statistical significance of the differences was determined by applying Student’s t-test at *p* < 0.05, *p* < 0.01 and *p* < 0.001.

## 5. Conclusions

This study investigated the relationship between ALDH1A1 expression and expression of drug transporters and ECM molecules in PAC- and TOP-resistant ovarian cancer cell lines. Regulation of *MDR1*/P-gp in PAC resistant cell lines seems to be ALDH1A1 independent. In contrast, changes of ALDH1A1 expression in TOP-resistant cell line resulted in BCRP, *MDR1*/P-gp and COL3A1 expression changes. In all cases, the same cell line was more resistant in 3D cell culture condition than in the 2D cell culture condition. The level of resistance in the 2D cell culture condition was related to the expression level of drug transporters. The main determinate of resistance in 3D spheroids seems to be the presence of different cells zones, the density of spheroids, and the capacity of the drug to diffuse into the cellular/ECM structure.

## Figures and Tables

**Table 1:** Summary of cell line resistance to PAC (paclitaxel) and TOP (topotecan) in two dimensional (2D) cell culture conditions.

Cell Line	PAC IC50 (ng/mL)	TOP IC50 (ng/mL)
W1	3.37 (3.18–3.62)	2.06 (1.94–2.18)
W1PR1	1671 *** (1526–1788)	-
W1PR1-C7	1342 ** ^ (1234–1451)	-
W1TR	3.96 (2.34–5.20)	111 ** (98.4–123)
W1TR-1p17	5.53 * (4.05–8.43)	7.18 *** ^^ (5.59–8.70)
W1TR-2p17	787 ** ^^ (682–843)	31.1 *** ^^ (27.5–34.9)

*—difference between drug sensitive (W1) and resistant cell line, the values were considered significant at * *p* < 0.05, ** *p* < 0.01, and *** *p* < 0.001; ^—difference between drug resistant cell line and cell line with changed ALDH1A1 expression (W1PR1-C7 vs. W1PR1; W1TR-1p17 and W1TR-2p17 vs. W1TR), the values were considered significant at ^ *p* < 0.05, and ^^ *p* < 0.01.

**Table 2:** Summary of cell line resistance to PAC (paclitaxel) and TOP (topotecan) in three-dimensional (3D) cell culture condition.

Cell Line	PAC IC50 (ng/mL)	TOP IC50 (ng/mL)
W1	11,883 (9548–15,833)	154 (65–279)
W1PR1	6022 ** (5302–7488)	-
W1PR1-C7	3971 *** (2933–5696) ^ *p* = 0.06	-
W1TR	6099 ** (5551–7353)	384 * (242–473)
W1TR-1p17	3283 *** ^^ (2831–4218)	814 *** ^^^ (629–1016)
W1TR-2p17	7183 * (5095–9544)	697 (406–797) *** ^

*—difference between drug sensitive (W1) and resistant cell line, the values were considered significant at * *p* < 0.05, ** *p* < 0.01, and *** *p* < 0.001; ^—difference between drug resistant cell line and cell line with changed ALDH1A1 expression; (W1PR1-C7 vs. W1PR1; W1TR-1p17 and W1TR-2p17 vs. W1TR), the values were considered significant at ^ *p* < 0.05, ^^ *p* < 0.01 and ^^^ *p* < 0.001.

**Table 3:** Oligonucleotide sequences used for RQ-PCR analysis.

Transcript	Sequence (5′-3′ Direction)	ENST Number http://www.ensembl.org accessed date: 10 September 2021	Product Size (bp)
*MDR1*	TGACAGCTACAGCACGGAAG TCTTCACCTCCAGGCTCAGT	00000265724	131 bp
*ALDH1A1*	GTTGTCAAACCAGCAGAGCA CTGTAGGCCCATAACCAGGA	00000165092	115 bp
*BCRP*	TTCGGCTTGCAACAACTATG TCCAGACACACCACGGATAA	00000237612	128 bp
*COL3A1*	AAGGTCCAGCTGGGATACCT CACCCTTTAATCCAGGAGCA	00000304636	105 bp
*GAPDH*	GAAGGTGAAGGTCGGAGTCA GACAAGCTTCCCGTTCTCAG	00000229239	199 bp
*β-actin*	TCTGGCACCACACCTTCTAC GATAGCACAGCCTGGATAGC	00000331789	169 bp
*HRPT1*	CTGAGGATTTGGAAAGGGTG AATCCAGCAGGTCAGCAAAG	00000298556	156 bp
*β2M*	CGCTACTCTCTCTTTCTGGC ATGTCGGATGGATGAAACCC	00000558401	133 bp

## Data Availability

Not applicable.

## References

[1] Webb P.M., Jordan S.J. (2017). Epidemiology of Epithelial Ovarian Cancer. Best Pr. Res. Clin. Obs. Gynaecol..

[2] Parmar M.K.B., Ledermann J.A., Colombo N., du Bois A., Delaloye J.-F., Kristensen G.B., Wheeler S., Swart A.M., Qian W., Torri V. (2003). Paclitaxel plus Platinum-Based Chemotherapy versus Conventional Platinum-Based Chemotherapy in Women with Relapsed Ovarian Cancer: The ICON4/AGO-OVAR-2.2 Trial. Lancet.

[3] Webber K., Friedlander M. (2017). Chemotherapy for Epithelial Ovarian, Fallopian Tube and Primary Peritoneal Cancer. Best Pract. Res. Clin. Obstet. Gynaecol..

[4] Norouzi-Barough L., Sarookhani M.R., Sharifi M., Moghbelinejad S., Jangjoo S., Salehi R. (2018). Molecular Mechanisms of Drug Resistance in Ovarian Cancer. J. Cell. Physiol..

[5] Leslie E.M., Deeley R.G., Cole S.P.C. (2005). Multidrug Resistance Proteins: Role of P-Glycoprotein, MRP1, MRP2, and BCRP (ABCG2) in Tissue Defense. Toxicol. Appl. Pharmacol..

[6] Wu C.-P., Calcagno A.M., Ambudkar S.V. (2008). Reversal of ABC Drug Transporter-Mediated Multidrug Resistance in Cancer Cells: Evaluation of Current Strategies. Curr. Mol. Pharm..

[7] Amawi H., Sim H.-M., Tiwari A.K., Ambudkar S.V., Shukla S., Liu X., Pan G. (2019). ABC Transporter-Mediated Multidrug-Resistant Cancer. Drug Transporters in Drug Disposition, Effects and Toxicity.

[8] Lehne G. (2000). P-Glycoprotein as a Drug Target in the Treatment of Multidrug Resistant Cancer. Curr. Drug Targets.

[9] Maliepaard M., van Gastelen M.A., de Jong L.A., Pluim D., Ruevekamp-Helmers M.C., Floot B.G.J., Schellens J.H.M. (1999). Overexpression of the BCRP/MXR/ABCP Gene in a Topotecan-Selected Ovarian Tumor Cell Line. 6. Cancer Res..

[10] Correia A.L., Bissell M.J. (2012). The Tumor Microenvironment Is a Dominant Force in Multidrug Resistance. Drug Resist. Updates.

[11] Cancer-Associated Fibroblasts in Tumor Microenvironment—Accomplices in Tumor Malignancy|Elsevier Enhanced Reader. https://reader.elsevier.com/reader/sd/pii/S0008874917302228?token=3F5F2EC8C1AA1D8ABBD54E618E3B9C0F1CBD41F79941B230D5E78EB2CF6534A367D905C2C59367DEE3A088B8BAAC2159&originRegion=eu-west-1&originCreation=20210914142221.

[12] Netti P.A., Berk D.A., Swartz M.A., Grodzinsky A.J., Jain R.K. (2000). Role of Extracellular Matrix Assembly in Interstitial Transport in Solid Tumors. Cancer Res..

[13] Grantab R., Sivananthan S., Tannock I.F. (2006). The Penetration of Anticancer Drugs through Tumor Tissue as a Function of Cellular Adhesion and Packing Density of Tumor Cells. Cancer Res..

[14] Wantoch von Rekowski K., König P., Henze S., Schlesinger M., Zawierucha P., Januchowski R., Bendas G. (2019). The Impact of Integrin-Mediated Matrix Adhesion on Cisplatin Resistance of W1 Ovarian Cancer Cells. Biomolecules.

[15] Wantoch von Rekowski K., König P., Henze S., Schlesinger M., Zawierucha P., Januchowski R., Bendas G. (2020). Insight into Cisplatin-Resistance Signaling of W1 Ovarian Cancer Cells Emerges MTOR and HSP27 as Targets for Sensitization Strategies. Int. J. Mol. Sci..

[16] Sterzyńska K., Klejewski A., Wojtowicz K., Świerczewska M., Andrzejewska M., Rusek D., Sobkowski M., Kędzia W., Brązert J., Nowicki M. (2018). The Role of Matrix Gla Protein (MGP) Expression in Paclitaxel and Topotecan Resistant Ovarian Cancer Cell Lines. Int. J. Mol. Sci..

[17] Senthebane D.A., Jonker T., Rowe A., Thomford N.E., Munro D., Dandara C., Wonkam A., Govender D., Calder B., Soares N.C. (2018). The Role of Tumor Microenvironment in Chemoresistance: 3D Extracellular Matrices as Accomplices. Int. J. Mol. Sci..

[18] Senthebane D.A., Rowe A., Thomford N.E., Shipanga H., Munro D., Mazeedi M.A.M.A., Almazyadi H.A.M., Kallmeyer K., Dandara C., Pepper M.S. (2017). The Role of Tumor Microenvironment in Chemoresistance: To Survive, Keep Your Enemies Closer. Int. J. Mol. Sci..

[19] Sterzyńska K., Klejewski A., Wojtowicz K., Świerczewska M., Nowacka M., Kaźmierczak D., Andrzejewska M., Rusek D., Brązert M., Brązert J. (2018). Mutual Expression of ALDH1A1, LOX, and Collagens in Ovarian Cancer Cell Lines as Combined CSCs- and ECM-Related Models of Drug Resistance Development. Int. J. Mol. Sci..

[20] Sterzyńska K., Kaźmierczak D., Klejewski A., Świerczewska M., Wojtowicz K., Nowacka M., Brązert J., Nowicki M., Januchowski R. (2019). Expression of Osteoblast-Specific Factor 2 (OSF-2, Periostin) Is Associated with Drug Resistance in Ovarian Cancer Cell Lines. Int. J. Mol. Sci..

[21] Klejewski A., Sterzyńska K., Wojtowicz K., Świerczewska M., Partyka M., Brązert M., Nowicki M., Zabel M., Januchowski R. (2017). The Significance of Lumican Expression in Ovarian Cancer Drug-Resistant Cell Lines. Oncotarget.

[22] Januchowski R., Świerczewska M., Sterzyńska K., Wojtowicz K., Nowicki M., Zabel M. (2016). Increased Expression of Several Collagen Genes Is Associated with Drug Resistance in Ovarian Cancer Cell Lines. J. Cancer.

[23] Moitra K., Lou H., Dean M. (2011). Multidrug Efflux Pumps and Cancer Stem Cells: Insights into Multidrug Resistance and Therapeutic Development. Clin. Pharmacol. Ther..

[24] Al-Alem L.F., Pandya U.M., Baker A.T., Bellio C., Zarrella B.D., Clark J., DiGloria C.M., Rueda B.R. (2019). Ovarian Cancer Stem Cells: What Progress Have We Made?. Int. J. Biochem. Cell Biol..

[25] Nwani N.G., Condello S., Wang Y., Swetzig W.M., Barber E., Hurley T., Matei D. (2019). A Novel ALDH1A1 Inhibitor Targets Cells with Stem Cell Characteristics in Ovarian Cancer. Cancers.

[26] Clevers H. (2011). The Cancer Stem Cell: Premises, Promises and Challenges. Nat. Med..

[27] Uddin M.H., Kim B., Cho U., Azmi A.S., Song Y.S. (2020). Association of ALDH1A1-NEK-2 Axis in Cisplatin Resistance in Ovarian Cancer Cells. Heliyon.

[28] ALDH1A1+ Ovarian Cancer Stem Cells Co-Expressing Surface Markers CD24, EPHA1 and CD9 Form Tumours In Vivo|Elsevier Enhanced Reader. https://reader.elsevier.com/reader/sd/pii/S0014482720302317?token=DD92BF30B6651F26F7E847502B429C4CE1C245C8A5E0414F6BBF46C22C8C8CF7E41FDC753F8B8734448BA207DE975CD0&originRegion=eu-west-1&originCreation=20210914145051.

[29] Inhibition of ALDH1A1 Activity Decreases Expression of Drug Transporters and Reduces Chemotherapy Resistance in Ovarian Cancer Cell Lines|Elsevier Enhanced Reader. https://reader.elsevier.com/reader/sd/pii/S1357272516301911?token=CB7EE6B98150BD6462E88D85FC4AA3B748FB5F72D780DFAD04CD00C30DD384310E7E36A1268C867CB80A624F6B06B7E0.

[30] The Stem Cell Markers Oct4A, Nanog and c-Myc Are Expressed in Ascites Cells and Tumor Tissue of Ovarian Cancer Patients|SpringerLink. https://link.springer.com/article/10.1007%2Fs13402-013-0142-8.

[31] Landen C.N., Goodman B., Katre A.A., Steg A.D., Nick A.M., Stone R.L., Miller L.D., Mejia P.V., Jennings N.B., Gershenson D.M. (2010). Targeting Aldehyde Dehydrogenase Cancer Stem Cells in Ovarian Cancer. Mol Cancer.

[32] Kaipio K., Chen P., Roering P., Huhtinen K., Mikkonen P., Östling P., Lehtinen L., Mansuri N., Korpela T., Potdar S. (2020). ALDH1A1-Related Stemness in High-Grade Serous Ovarian Cancer Is a Negative Prognostic Indicator but Potentially Targetable by EGFR/MTOR-PI3K/Aurora Kinase Inhibitors. J. Pathol..

[33] Sherman-Baust C.A., Weeraratna A.T., Rangel L.B.A., Pizer E.S., Cho K.R., Schwartz D.R., Shock T., Morin P.J. (2003). Remodeling of the Extracellular Matrix through Overexpression of Collagen VI Contributes to Cisplatin Resistance in Ovarian Cancer Cells. Cancer Cell.

[34] Morin P.J. (2003). Drug Resistance and the Microenvironment: Nature and Nurture. Drug Resist. Updates.

[35] Helleman J., Jansen M.P.H.M., Span P.N., van Staveren I.L., Massuger L.F.A.G., Gelder M.E.M., Sweep F.C.G.J., Ewing P.C., van der Burg M.E.L., Stoter G. (2006). Molecular Profiling of Platinum Resistant Ovarian Cancer. Int. J. Cancer.

[36] Chien J., Kuang R., Landen C., Shridhar V. (2013). Platinum-Sensitive Recurrence in Ovarian Cancer: The Role of Tumor Microenvironment. Front. Oncol..

[37] Januchowski R., Zawierucha P., Andrzejewska M., Ruciński M., Zabel M. (2013). Microarray-Based Detection and Expression Analysis of ABC and SLC Transporters in Drug-Resistant Ovarian Cancer Cell Lines. Biomed. Pharmacother..

[38] Januchowski R., Wojtowicz K., Sujka-Kordowska P., Andrzejewska M., Zabel M. (2013). MDR Gene Expression Analysis of Six Drug-Resistant Ovarian Cancer Cell Lines. BioMed Res. Int..

[39] Januchowski R., Zawierucha P., Ruciński M., Zabel M. (2014). Microarray-Based Detection and Expression Analysis of Extracellular Matrix Proteins in Drug-resistant Ovarian Cancer Cell Lines. Oncol. Rep..

[40] Sterzyńska K., Klejewski A., Wojtowicz K., Świerczewska M., Nowicki M., Brązert J., Januchowski R. (2018). Myotilin, a New Topotecan Resistant Protein in Ovarian Cancer Cell Lines. J. Cancer.

[41] Nowacka M., Sterzynska K., Andrzejewska M., Nowicki M., Januchowski R. (2021). Drug Resistance Evaluation in Novel 3D In Vitro Model. Biomed. Pharmacother..

[42] Ozben T. (2006). Mechanisms and Strategies to Overcome Multiple Drug Resistance in Cancer. FEBS Lett..

[43] Pignata S., C Cecere S., Du Bois A., Harter P., Heitz F. (2017). Treatment of Recurrent Ovarian Cancer. Ann. Oncol..

[44] Tirino V., Desiderio V., Paino F., De Rosa A., Papaccio F., La Noce M., Laino L., De Francesco F., Papaccio G. (2013). Cancer Stem Cells in Solid Tumors: An Overview and New Approaches for Their Isolation and Characterization. FASEB J..

[45] The Role of Aldehyde Dehydrogenase (ALDH) in Cancer Drug Resistance|Elsevier Enhanced Reader. https://reader.elsevier.com/reader/sd/pii/S0753332213000590?token=4EA5C8DCA1128518F46D9140972528C5B0C4F11C7D449671190A08CED4E87DB0F507FD83E1D48578A546C0197167A6D3&originRegion=eu-west-1&originCreation=20211103135350.

[46] Steg A.D., Bevis K.S., Katre A.A., Ziebarth A., Dobbin Z.C., Alvarez R.D., Zhang K., Conner M., Landen C.N. (2012). Stem Cell Pathways Contribute to Clinical Chemoresistance in Ovarian Cancer. Clin. Cancer Res..

[47] Liu S., Liu C., Min X., Ji Y., Wang N., Liu D., Cai J., Li K. (2013). Prognostic Value of Cancer Stem Cell Marker Aldehyde Dehydrogenase in Ovarian Cancer: A Meta-Analysis. PLoS ONE.

[48] Aldehyde Dehydrogenase in Combination with CD133 Defines Angiogenic Ovarian Cancer Stem Cells That Portend Poor Patient Survival|Cancer Research. https://cancerres.aacrjournals.org/content/71/11/3991.long.

[49] ALDH1-Bright Epithelial Ovarian Cancer Cells Are Associated with CD44 Expression, Drug Resistance, and Poor Clinical Outcome-ClinicalKey. https://www.clinicalkey.com/#!/content/playContent/1-s2.0-S0002944011010790?returnurl=https:%2F%2Flinkinghub.elsevier.com%2Fretrieve%2Fpii%2FS0002944011010790%3Fshowall%3Dtrue&referrer=.

[50] Aldehyde Dehydrogenase 1A1 (ALDH1A1) Expression by Immunohistochemistry Is Associated with Chemo-Refractoriness in Patients with High-Grade Ovarian Serous Carcinoma|Elsevier Enhanced Reader. https://reader.elsevier.com/reader/sd/pii/S0046817717302976?token=8A99D1BB1D312814F964D405FC5A06B2279D90C7336AA2E75172655CF61CBA3A8FD1E98E87FAE6120CFC613D720977EC&originRegion=eu-west-1&originCreation=20211013083007.

[51] Ayub T.H., Keyver-Paik M.-D., Debald M., Rostamzadeh B., Thiesler T., Schröder L., Barchet W., Abramian A., Kaiser C., Kristiansen G. (2015). Accumulation of ALDH1-Positive Cells after Neoadjuvant Chemotherapy Predicts Treatment Resistance and Prognosticates Poor Outcome in Ovarian Cancer. Oncotarget.

[52] Han X., Du F., Jiang L., Zhu Y., Chen Z., Liu Y., Hong T., Wang T., Mao Y., Wu X. (2013). A2780 Human Ovarian Cancer Cells with Acquired Paclitaxel Resistance Display Cancer Stem Cell Properties. Oncol. Lett..

[53] Hilton J. (1984). Role of Aldehyde Dehydrogenase in Cyclophosphamide-Resistant L1210 Leukemia. Cancer Res..

[54] Moreb J., Zucali J.R., Zhang Y., Colvin M.O., Gross M.A. (1992). Role of Aldehyde Dehydrogenase in the Protection of Hematopoietic Progenitor Cells from 4-Hydroperoxycyclophosphamide by Inter Leukin IÃŸand Tumor Necrosis Factor. Cancer Res..

[55] Alison M.R., Lin W.-R., Lim S.M.L., Nicholson L.J. (2012). Cancer Stem Cells: In the Line of Fire. Cancer Treat. Rev..

[56] Yang Y., Zhou W., Xia J., Gu Z., Wendlandt E., Zhan X., Janz S., Tricot G., Zhan F. (2014). NEK2 Mediates ALDH1A1-Dependent Drug Resistance in Multiple Myeloma. Oncotarget.

[57] Kim D., Choi B., Ryoo I., Kwak M.-K. (2018). High NRF2 Level Mediates Cancer Stem Cell-like Properties of Aldehyde Dehydrogenase (ALDH)-High Ovarian Cancer Cells: Inhibitory Role of All-Trans Retinoic Acid in ALDH/NRF2 Signaling. Cell Death Dis..

[58] Januchowski R., Zawierucha P., Ruciński M., Andrzejewska M., Wojtowicz K., Nowicki M., Zabel M. (2014). Drug Transporter Expression Profiling in Chemoresistant Variants of the A2780 Ovarian Cancer Cell Line. Biomed. Pharmacother..

[59] Januchowski R., Wojtowicz K., Andrzejewska M., Zabel M. (2014). Expression of MDR1 and MDR3 Gene Products in Paclitaxel-, Doxorubicin- and Vincristine-Resistant Cell Lines. Biomed. Pharmacother..

[60] Vanhoefer U., Müller M.R., Hilger R.A., Lindtner B., Klaassen U., Schleucher N., Rustum Y.M., Seeber S., Harstrick A. (1999). Reversal of MDR1-Associated Resistance to Topotecan by PAK-200S, a New Dihydropyridine Analogue, in Human Cancer Cell Lines. Br. J. Cancer.

[61] Januchowski R., Sterzyńska K., Zaorska K., Sosińska P., Klejewski A., Brązert M., Nowicki M., Zabel M. (2016). Analysis of MDR Genes Expression and Cross-Resistance in Eight Drug Resistant Ovarian Cancer Cell Lines. J. Ovarian Res..

[62] Weiswald L.-B., Bellet D., Dangles-Marie V. (2015). Spherical Cancer Models in Tumor Biology. Neoplasia.

[63] Hamilton G. (1998). Multicellular Spheroids as an in Vitro Tumor Model. Cancer Lett..

[64] Erlanson M., Daniel-Szolgay E., Carlsson J. (1992). Relations between the Penetration, Binding and Average Concentration of Cytostatic Drugs in Human Tumour Spheroids. Cancer Chemother. Pharmacol..

[65] Wang J., Lu Z., Gao Y., Wientjes M.G., Au J.L.-S. (2011). Improving Delivery and Efficacy of Nanomedicines in Solid Tumors: Role of Tumor Priming. Nanomedicine.

[66] Pommier Y., Sun Y., Huang S.N., Nitiss J.L. (2016). Roes of Eukaryotic Topoisomerases in Transcription, Replication and Genomic Stability. Nat. Rev. Mol. Cell Biol..

[67] Staker B.L., Hjerrild K., Feese M.D., Behnke C.A., Burgin A.B., Stewart L. (2002). The Mechanism of Topoisomerase I Poisoning by a Camptothecin Analog. Proc. Natl. Acad. Sci. USA.

[68] Uhm J.H., Dooley N.P., Kyritsis A.P., Rao J.S., Gladson C.L. (1999). Vitronectin, a Glioma-Derived Extracellular Matrix Protein, Protects Tumor Cells from Apoptotic Death. Clin. Cancer Res..

[69] Stylianopoulos T., Diop-Frimpong B., Munn L.L., Jain R.K. (2010). Diffusion Anisotropy in Collagen Gels and Tumors: The Effect of Fiber Network Orientation. Biophys. J..

[70] Nederman T., Carlsson J. (1984). Penetration and Binding of Vinblastine and 5-Fluorouracil in Cellular Spheroids. Cancer Chemother. Pharmacol..

[71] Imamura Y., Mukohara T., Shimono Y., Funakoshi Y., Chayahara N., Toyoda M., Kiyota N., Takao S., Kono S., Nakatsura T. (2015). Comparison of 2D- and 3D-Culture Models as Drug-Testing Platforms in Breast Cancer. Oncol. Rep..

[72] Myungjin Lee J., Mhawech-Fauceglia P., Lee N., Cristina Parsanian L., Gail Lin Y., Andrew Gayther S., Lawrenson K. (2013). A Three-Dimensional Microenvironment Alters Protein Expression and Chemosensitivity of Epithelial Ovarian Cancer Cells in Vitro. Lab. Invest..

[73] Comparison of 2D and 3D Cell Culture Models for Cell Growth, Gene Expression and Drug Resistance|Elsevier Enhanced Reader. https://reader.elsevier.com/reader/sd/pii/S0928493119305934?token=A81072D392015C0F3853335103228D48E9C60158BAE61A69162483955AB135BBAA3EB87B3FA7A19190F78188B23943EC&originRegion=eu-west-1&originCreation=20211103150017.

